# Alternate Estrogen Receptors Promote Invasion of Inflammatory Breast Cancer Cells via Non-Genomic Signaling

**DOI:** 10.1371/journal.pone.0030725

**Published:** 2012-01-25

**Authors:** Kazufumi Ohshiro, Arnold M. Schwartz, Paul H. Levine, Rakesh Kumar

**Affiliations:** 1 Department of Biochemistry and Molecular Biology, The George Washington University Medical Center, Washington, D.C., United States of America; 2 Department of Pathology, The George Washington University Medical Center, Washington, D.C., United States of America; 3 Department of Epidemiology and Biostatistics, The George Washington University Medical Center, Washington, D.C., United States of America; Wayne State University, United States of America

## Abstract

Although Inflammatory Breast Cancer (IBC) is a rare and an aggressive type of locally advanced breast cancer with a generally worst prognosis, little work has been done in identifying the status of non-genomic signaling in the invasiveness of IBC. The present study was performed to explore the status of non-genomic signaling as affected by various estrogenic and anti-estrogenic agents in IBC cell lines SUM149 and SUM190. We have identified the presence of estrogen receptor α (ERα) variant, ERα36 in SUM149 and SUM190 cells. This variant as well as ERβ was present in a substantial concentration in IBC cells. The treatment with estradiol (E2), anti-estrogenic agents 4-hydroxytamoxifen and ICI 182780, ERβ specific ligand DPN and GPR30 agonist G1 led to a rapid activation of p-ERK1/2, suggesting the involvement of ERα36, ERβ and GPR30 in the non-genomic signaling pathway in these cells. We also found a substantial increase in the cell migration and invasiveness of SUM149 cells upon the treatment with these ligands. Both basal and ligand-induced migration and invasiveness of SUM149 cells were drastically reduced in the presence of MEK inhibitor U0126, implicating that the phosphorylation of ERK1/2 by MEK is involved in the observed motility and invasiveness of IBC cells. We also provide evidence for the upregulation of p-ERK1/2 through immunostaining in IBC patient samples. These findings suggest a role of non-genomic signaling through the activation of p-ERK1/2 in the hormonal dependence of IBC by a combination of estrogen receptors. These findings only explain the failure of traditional anti-estrogen therapies in ER-positive IBC which induces the non-genomic signaling, but also opens newer avenues for design of modified therapies targeting these estrogen receptors.

## Introduction

Inflammatory Breast Cancer (IBC) is a rare and aggressive form of locally advanced breast cancer affecting approximately 1–6% of breast cancer patients in the United States [1]. It is reported by the National Cancer Institute's Surveillance, Epidemiology, and End Results (SEER) that while the incidence of normal breast cancer has been steadily decreasing, the incidence of IBC continues to be increasing. SEER further reports that the overall survival rate of IBC patients is significantly lower than non-IBC stage III breast cancer [2]. The aggressive characteristic of IBC enables its malignant cells to invade the dermal lymphatics of the breast, causing the accumulation of fluids within lymphatic vessels and the subsequent edematous red swelling of the breast [3]. By its ability to metastasize rapidly, most IBC tumors are characterized as stage IIIB at the time of detection [4]. These characteristics coupled with the relatively poor prognosis makes IBC one of the most deadly carcinomas.

Since the term IBC was coined by Lee and Tannebaum in 1924, there has been an extensive search for an effective way to treat this disease. Although many groups have reported that the prognosis of the disease can be improved by multimodality treatment, improvement in the overall survival rate is still poor due to the lack of a major molecular signature [5]–[7]. Several studies have found that an alteration in the levels of molecules such as RhoC, WISP-3 and Caveolin contribute significantly to IBC progression, but an IBC-specific therapy still remains elusive [8]–[11].

IBC is predominantly negative for estrogen receptor α (ERα) and progesterone receptor (PR) and positive for human epidermal growth factor receptor 2 (HER2) [12]. The absence of ERα makes it difficult to treat IBC with traditional antiestrogens such as tamoxifen. In addition to the wild-type ERα, several groups have provided evidence for the existence of ERα splice variants. Of the splice variants, ERα36 has been relatively well studied. ERα36 is translated from a transcript initiated by a previously unidentified promoter in the first intron of ERα gene and lacks both AF-1 and AF-2 transactivation domains present in ERα [13]. ERα36 was expressed in both ERα positive and negative breast carcinomas and its expression was reduced with the presence of ERα [14]. Furthermore, ERα36 has been shown to be capable of supporting the non-genomic signaling pathways such as ERK and Akt in endometrial carcinomas and HEK293 cells [15], [16]. The search for an alternate estrogen receptor led to an identification of a novel G-protein coupled receptor, GPR30 that localized in the plasma membrane, the cytoplasm and the endoplasmic reticulum [17]–[19]. GPR30 is responsible for its rapid activation of non-genomic signaling pathway and is involved in the biological processes including migration, proliferation, adhesion, gene regulation and invasion of the cancer cells [18]–[21]. Interestingly, the classical anti-estrogens such as tamoxifen and ICI 182780 are known to act as agonists for ERα36 and GPR30. Therefore, these alternate estrogen receptors are considered to contribute to the tamoxifen resistance in a variety of tumors [22], [23].

Stimulation of the estrogenic non-genomic signaling components such as PI3K, Akt and ERK contributes to alterations in the downstream effectors in a cell-type-specific manner and causes rapid physiological responses in target tissues [24]. In spite of the immense importance of non-genomic signaling pathway in other tumors, little research has been done on its role in IBC. In the present study, we discovered the presence of ERα36 and ERβ in IBC cells and also found an evidence for the possible contribution of these variants in the stimulation of p-ERK1/2 in response to treatment with agonists and antagonists. Furthermore, we found that this stimulation of p-ERK1/2 leaded to an enhanced migration and invasiveness of IBC cells.

## Results

### Expression status of estrogen receptors in SUM149 and SUM190 cells

**Figure 1:**
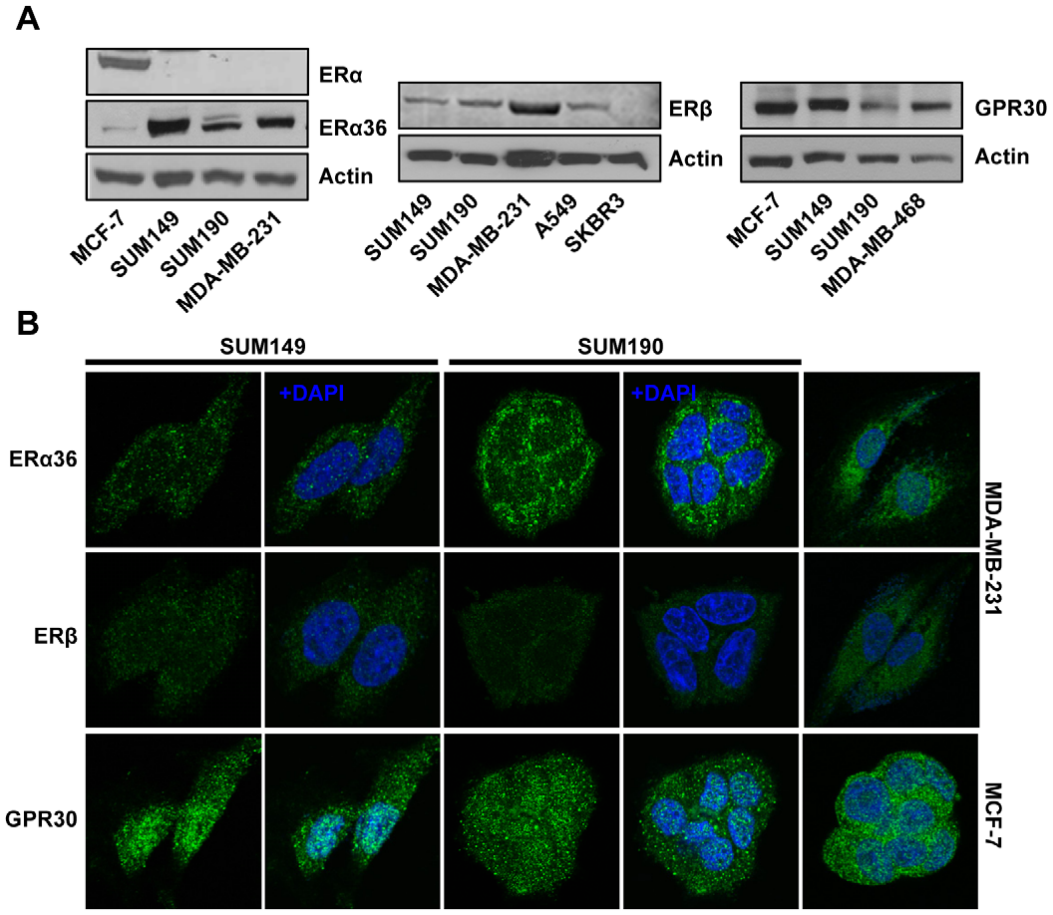
Estrogen receptor expression profiling in IBC cell lines SUM149 and SUM190. (A) Western blot analysis of ERα36, ERβ and GPR30 (B) Immunofluorescent localization of ERα36, ERβ and GPR30. Estrogen receptors (green) and DNA (blue) in these cells.

The full-length ERα was negative in the IBC cell lines SUM149 and SUM190 (Fig. 1A), while ERα alternative splicing variant ERα36 was substantially expressed in the both cell lines (Fig. 1A). ERβ was detectable in both SUM149 and SUM190 cells. As expected, MDA-MB-231 and SKBR3 cells were positive and negative for ERβ expression, respectively. GPR30 were also detected in both SUM149 and SUM190 as well as in GPR30-positive cell lines MCF-7 and MDA-MB-468 (Fig.1A). Immunoflourescent labeling followed by visualization through scanning confocal microscopy revealed that while ERα36 was predominantly localized in the cytoplasmic compartment in both SUM149 and SUM190 cells, ERβ was detected with low expression levels in the both cytoplasm and nucleus of these cell lines (Fig. 1B). GPR30 was present in both the cytoplasmic and nuclear compartment in these cells.

### Rapid activation of p-ERK1/2 by estrogen receptor ligands in SUM149 and SUM190 cells

**Figure 2:**
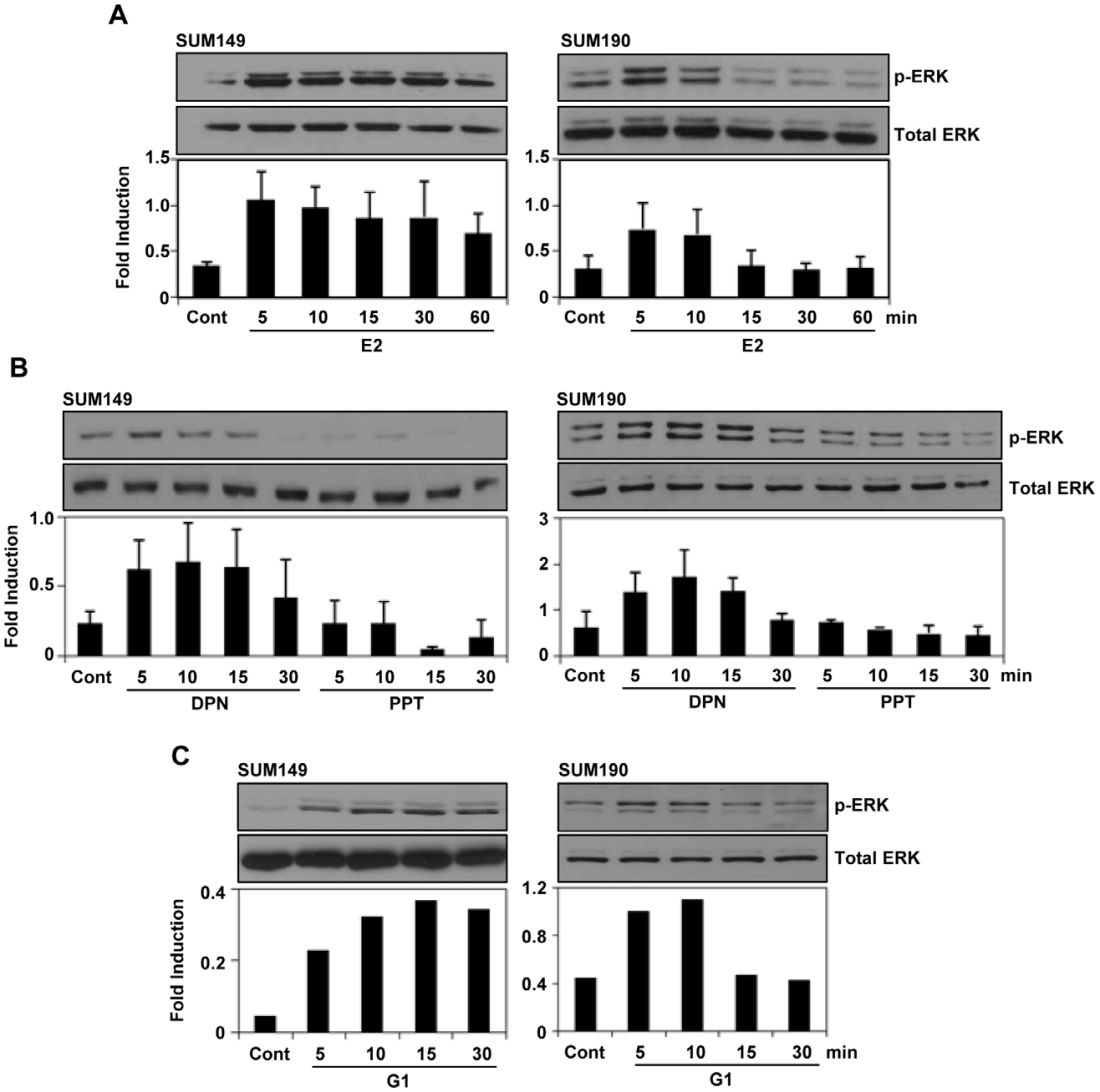
Estrogen receptor ligands induced ERK1/2 phosphorylation in SUM149 and SUM190 cells. (A–C) The cells were maintained in 5% DCC for 48 hours and then treated with E2 (10 nM), DPN (10 nM) or G1 (10 nM) for the indicated times and then ERK1/2 phosphorylation was analyzed by western blotting. The expression level of total ERK was analyzed for normalization. Error bars indicate standard deviation.

The localization of the estrogen receptors in the cytoplasmic compartment presented a possibility of the involvement of non-genomic signaling in these cells. To investigate this possibility, SUM149 and SUM190 cells were treated with E2 (10 nM) after maintained in 5% DCC medium for 48 hrs. We noticed a rapid activation of p-ERK1/2 within 5 min of post E2 treatment in SUM149 and SUM190 cells (Fig. 2A). However, the phosphorylation of the other MAPKs JNK and p38 were hardly activated by E2 treatment in SUM149 cells (supplementary Fig. S1). In addition, Akt phosphorylation did not increased upon E2 treatment. Interestingly, we also found a rapid activation of p-ERK1/2 in these cells upon treatment with 10 nM of ERβ specific ligand DPN and GPR30-specific agonist G1 (Fig. 2B, C). However, there was no p-ERK1/2 activation upon the treatment with 10 nM of ERα-specific ligand PPT (Fig. 2B).

**Figure 3:**
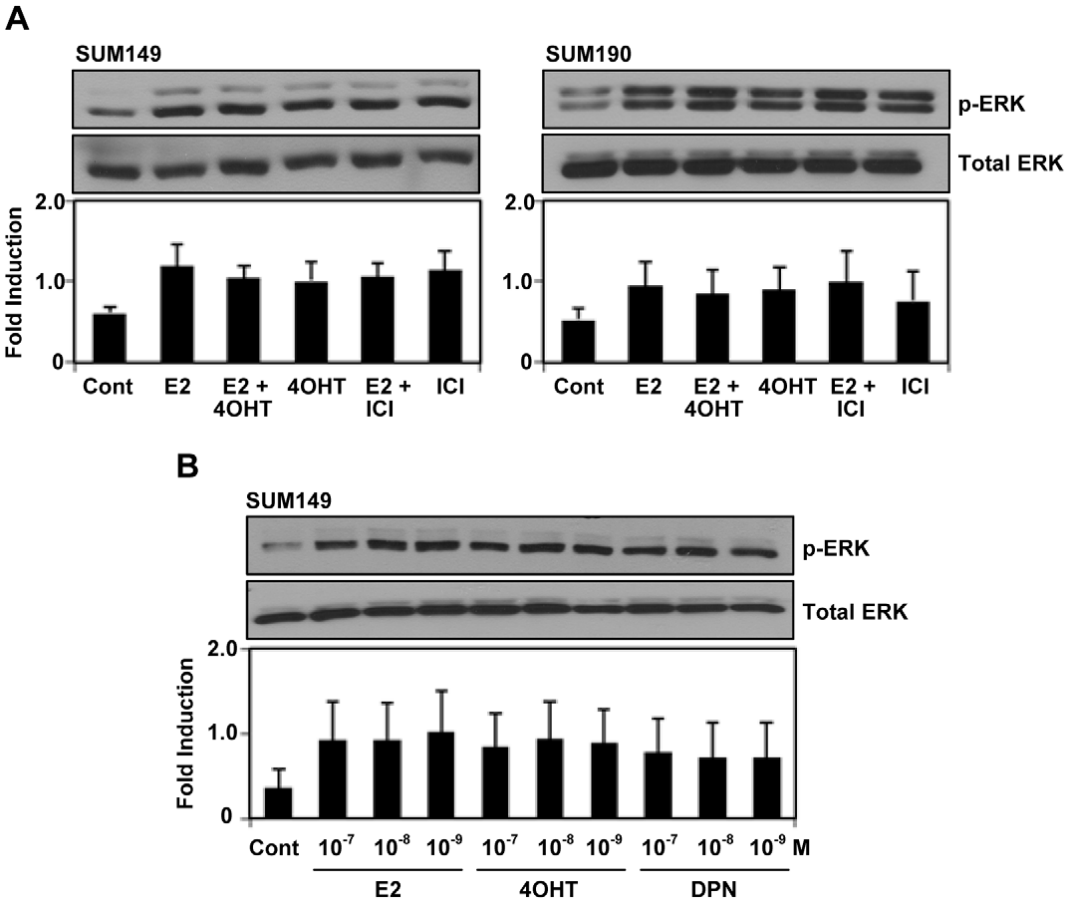
Effect of traditional antiestrogen treatment in SUM149 and SUM190 cells. (A) Effect of 4-hyroxytamoxifen (4OHT) and ICI 182780 (ICI) on E2-induced ERK1/2 phosphorylation. The cells were maintained in 5% DCC for 48 hours and then pretreated with or without ICI (100 nM) or 4OHT (100 nM) for1 hour before the treatment with or without E2 (10 nM). ERK1/2 phosphorylation was analyzed by western blotting. (B) Effect of dose dependent treatment of E2, 4OHT and DPN in SUM149 cells. The cells were maintained in 5% DCC for 48 hrs and then E2, 4OHT or DPN were treated at three concentrations (1, 10, 100 nM) of for 5 min. Error bars indicate standard deviation.

**Figure 4:**
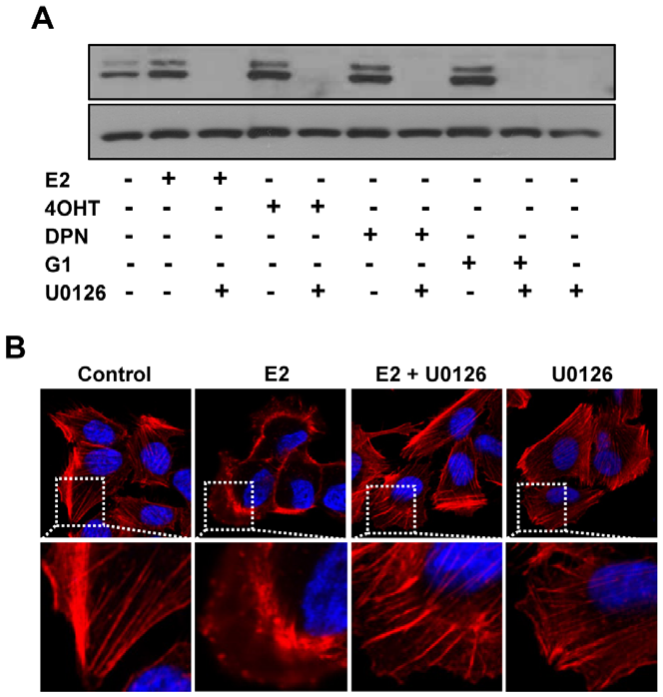
Effect of MEK inhibitor U0126 on p-ERK1/2 activation and cytoskeletal remodeling induced by estrogen receptor ligands and antiestrogens. (A) Blocking of E2, DPN, 4OHT or G1 induced ERK1/2 phosphorylation in SUM149 cells by U0126. The cells were maintained in 5% DCC for 48 hours and then pretreated with or without U0126 (20 µM) for 15 min before treatment with or without E2 (10 nM), DPN (10 nM), 4OHT (10 nM) and G1 (10 nM) for 5 min. ERK1/2 phosphorylation was analyzed by western blotting. (B) Blocking of E2-induced cytoskeletal change in SUM149 cells by U0126. The cells were maintained for 48 hours in 5% DCC and then pretreated with U0126 (20 µM) before the treatment with or without E2 (10 nM) for 5 min. The cells were fixed and labeled with fluorescently conjugated phalloidin (for filamentous actin, red) and DAPI (for DNA). White-dotted areas in upper panels were magnified in lower panels.

The anti-estrogens 4OHT and ICI are known to inhibit the response of E2 mediated by ERα. Next, we tested whether these anti-estrogens could compromise the activation of p-ERK1/2 by E2 treatment. Interestingly, we found that the treatment of IBC cells with OHT or ICI, alone or in combination with E2 potentiated the magnitude of p-ERK1/2 stimulation by E2 (10 nM) or DPN (10 nM) (Fig. 3A). In addition, all of E2, 4OHT or DPN at 10^−7^ to 10^−9^M activated p-ERK1/2 (Fig. 3B). Pretreatment of SUM149 cells with a specific MEK inhibitor U0126 blocked the activation of p-ERK1/2 in response to stimulation with E2, 4OHT, DPN or G1 in SUM149 cells (Fig. 4A). Confocal microscopic analysis showed that E2 treatment induced cytoskeletal reorganization in SUM149 cells as revealed by actin staining (Fig. 4B), indicating that the cell mobility might be positively regulated by E2, which could be effectively blocked by the pretreatment of MEK inhibitor U0126.

### Activation of p-ERK1/2 promotes motility and invasiveness of SUM149 cells

**Figure 5:**
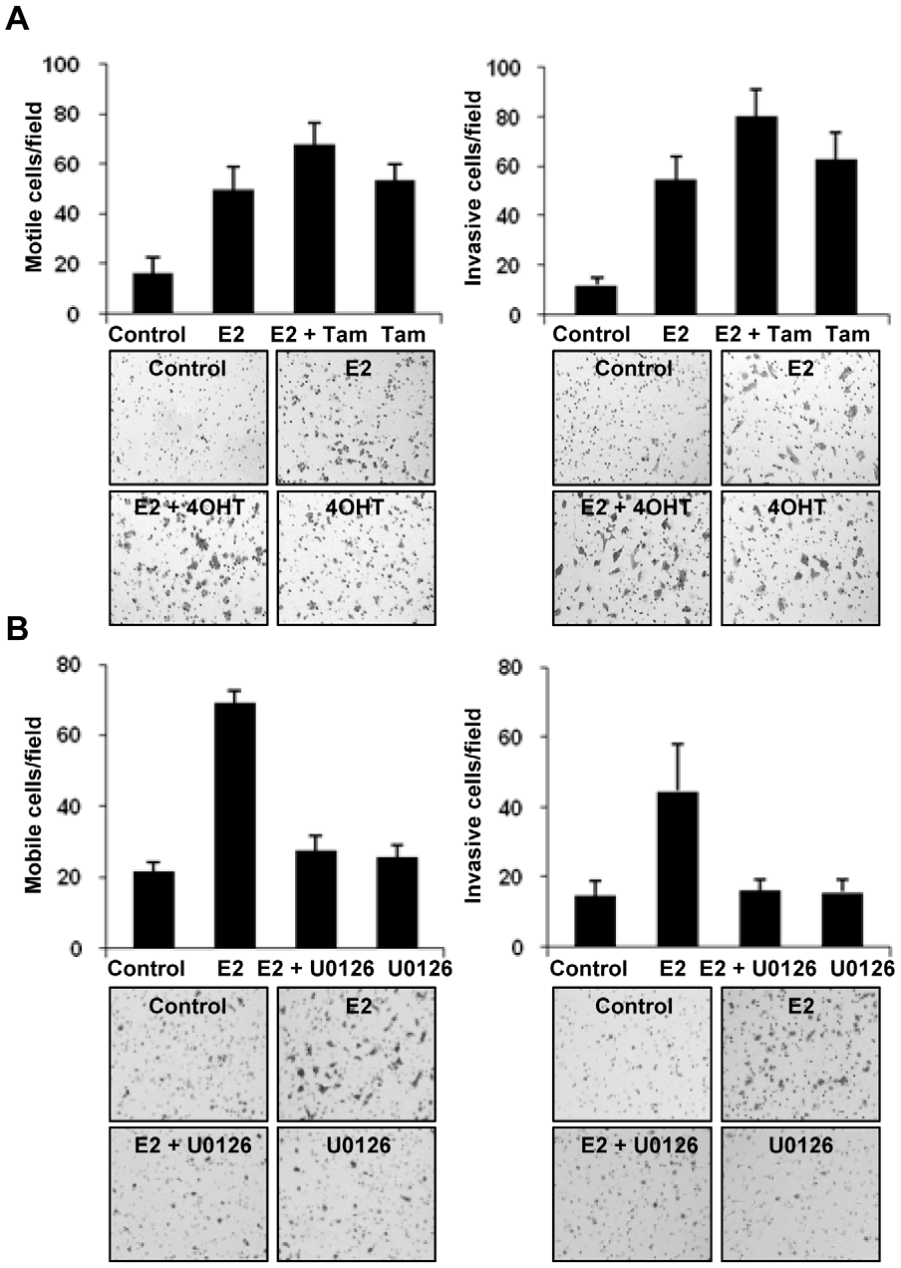
E2-induced SUM149 cell migration and invasion. (A) For migration, the cells (2 × 10^4^ cells) were added on the lower side of a Boyden chamber and incubated for 14 hours. Then the cells that passed through the filters were fixed, stained, and counted (left). For invasion, the cells (1 × 10^5^ cells) were added to the lower side of a matrigel coated Boyden chamber and incubated for 20 hours. Then the cells that invaded through the matrigel were fixed stained and counted (right). (B) The E2-induced migration and invasion was blocked by treatment with U0126. Error bars indicate standard deviation.

Downstream signaling through the activation of p-ERK1/2 has been implicated in increasing the proliferation and motility in numerous cancer cell models. Therefore, we initially investigated whether E2 could stimulate IBC cell proliferation. The treatment with E2 at different doses (0.1–10 nM) had no effect on the cell proliferation (Supplementary Fig. S2). We then performed a Boyden chamber migration and invasion assay. The results showed that the treatment with E2 (10 nM) or/and 4OHT (100 nM) drastically increased the ability of the SUM149 cells to migrate and invade into the lower side of the well through the matrigel (Fig. 5A). To confirm if the observed increase in the invasiveness of SUM149 cells is mediated by the ERK pathway, we treated the cells with U0126 (20 µM) alone and in combination with E2 (10 nM), and a substantial reduction in the migration and invasion by inclusion of MEK inhibitor U0126 (Fig. 5B).

### Activation of p-ERK1/2 in IBC tissues

**Figure 6:**
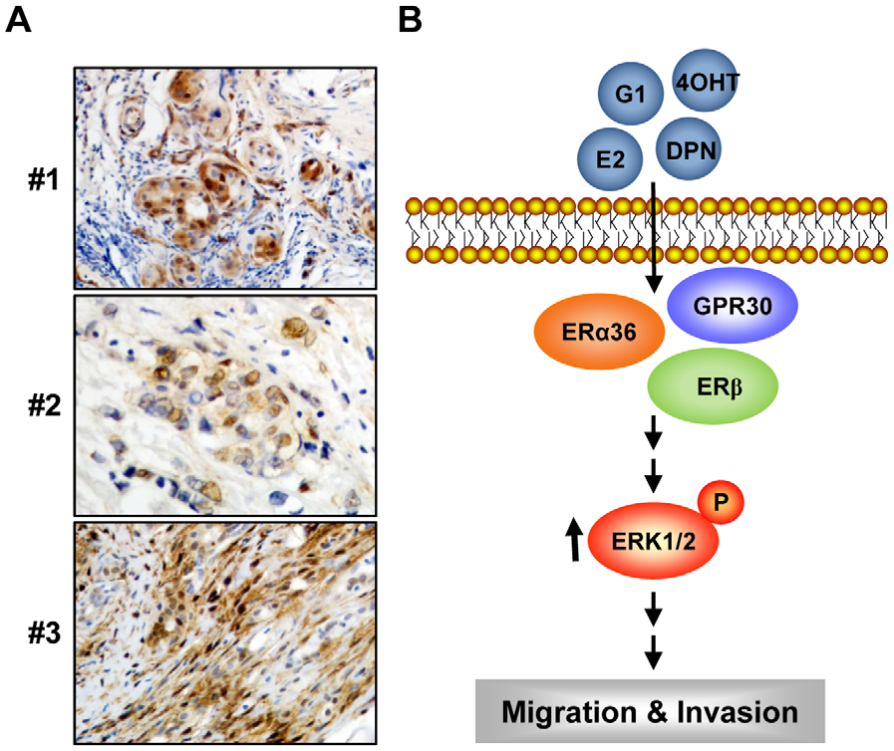
p-ERK1/2 expression of in IBC tissues. (A) The expression of p-ERK1/2 was tested in tumor tissue samples from patients with IBC using the rabbit anti-p-ERK1/2 antibody. (B) Proposed working model for the stimulation of estrogen receptor ligand-induced non-genomic signaling pathway via p-ERK1/2 activation in IBC cells. Estrogen receptor ligand rapidly activates p-ERK1/2 phosphorylation, leading to the activation of estrogen non-genomic signaling including cell migration and invasiveness in inflammatory breast cancer.

To validate the proof-of-principle evidence of p-ERK1/2 in IBC tumors, we examined immunohistochemically p-ERK1/2 expression in the tissues from five IBC patients using anti-p-ERK1/2 antibody (Fig. 6A). Three of five tissues displayed nuclear immunostaining of p-ERK1/2 with strong (#1, #3) and the moderate (#2) intensity, and two cases were negative or weak staining.

## Discussion

ERα is known to be absent in many cases of IBC and its absence is associated with poor clinical consequences [25]–[27]. In this study, we did not detect ERα in both IBC cell line SUM149 and SUM190, however, we identified the presence of ERα variant, ERα36. ERα36 was found to be mainly present in plasma membrane and cytoplasm and to be involved in estrogen non-genomic signaling [15], [28]. Subsequently, ERα36 has been reported to be potentially involved in the non-genomic signaling in several cancers including endometrial and gastric cancers [16], [29]. Recently, E2 treatment has been reported to enhance the cell proliferation of ERα negative cell line, MDA-MB-231 and MDA-MB-468 through EGFR/Src/ERK signaling pathway [30], while the cell proliferation of SUM149 and SUM190 were not affected by E2 treatment, indicating no stimulation of estrogen mitogenic signaling. However, the migration and invasion were promoted by E2 treatment in both SUM149 and SUM190 cells, revealing that the non-genomic estrogen signaling might be associated with the aggressive characteristic of IBC cells to invade into lymph node.

The involvement of GPR30 in the non-genomic signaling is found in both ER-positive and -negative breast cancer cells [18]. The activation of p-ERK1/2 by the treatment of GPR30-specific agonist G1 also suggested the involvement of GPR30 in the non-genomic signaling cascade in IBC cells. The fact that GPR30 localized in the nucleus than in the cytoplasm in the IBC cells might suggest its lower contribution in activating non-genomic signaling by the GPR30 pathway. However, it has been shown that the nuclear localized GPR30 could also participate in the non-genomic signaling [31]. Kang et al. have shown that G1 is also able to stimulate ERα36 [23]. Furthermore, they have shown that the non-genomic signaling pathway through p-ERK1/2 is directly mediated by ERα36 but not by GPR30, because the activities of GPR30 promoted by estrogen were due to its ability to induce ERα36 expression. However, it remains possible that both receptors can play a crucial role in the non-genomic signaling pathway. Although we did not investigate whether G1 treatment upregulated the expression of ERα36 in these cells, there might be another layer of cross-talk between the GPR30 and ERα36 rather than the regulation of ERα36 protein expression by GPR30 as the primary mechanism of the noted non-genomic signaling.

In the IBC cells, the involvement of ERs in the non-genomic signaling seems to be more complicated due to the expression of ERβ in addition to the expression of ERα36 and GPR30. The involvement of ERβ in the non-genomic signaling pathway has been shown in several cancer cell lines [32], [33]. The rapid activation of p-ERK1/2 by treatment with ERβ-specific ligand DPN in IBC cells suggested a possible contribution of ERβ in the non-genomic signaling. Recent data from the uterine carcinosarcoma suggests a possible combined role of ERβ and GPR30 in the non-genomic signaling [34]. In addition, co-ordinated regulation of GPR30 and ERβ in endometrial cells upon E2 treatment also leads to an increased expression of GPR30 mRNA through ERβ [35]. Taken together, these data provide us the possibility that all of GPR30, ERα36 and ERβ might be involved in the non-genomic signaling through a net-work of cross-talks among ERs in IBC cells. However, further studies must investigate these possibilities.

We also found that 4OHT and the pure antiestrogen ICI also failed to block the E2-mediated activation of p-ERK1/2 in IBC cells. The antiestrogens including tamoxifen, 4OHT and ICI have been reported to act as agonists for ERα36 and GPR30 in a variety of cancer cells [15], [36]–[39]. One of potential mechanisms which causes antiestrogen-resistant in some breast cancers could be the stimulation of anti-estrogen signaling pathways via ERα36 [15]. Since expression of ER/PR is generally lower in IBC cells [25], [40], [41], such agents are not used for IBC therapeutics. Our findings presented here raise a new possibility that ERα variant ERα36, ERβ and GPR30 might be potentially targeted for IBC therapeutic. In this context, a recent study has shown that estriol could act as a GPR30 antagonist by inhibiting the GPR30-mediated activation of p-ERK1/2 in ERα-negative breast cancer cells [42]. Search and discovery for the other antiestrogens and antagonists including estriol to block non-genomic signaling components through ERs could provide novel insights into the therapeutic strategy for IBC patients. In addition, we have shown IBC non-genomic signaling function including the migration and the invasiveness of SUM149 cells via estrogen- and antiestrogen-mediated p-ERK1/2 activation (Figure 6B). Our present study suggests that in addition to ERs-targeted agents, the drugs which specifically inhibit ERK1/2 might prove to be effective in IBC, either as a single agent or in combination with targeted therapeutics to evolve an effective anti-IBC therapeutic strategy.

## Materials and Methods

### Cell culture and reagents

SUM149 and SUM190 were obtained from Asterand, plc (Detroit, MI) and maintained in Ham-F-12 (1∶1) medium supplemented with 5% Fetal Bovine Serum (FBS), 5 µg/ml insulin, 1 µg/ml hydrocortisone and 10 mM HEPES. SUM149 cell line is obtained from the primary ductal carcinoma of the breast from a patient with a locally advanced disease while SUM190 is obtained from poorly differentiated inflammatory breast carcinoma. Breast cancer cell lines MDA-MB-231, MDA-MB-468 and MCF-7 were purchased from the American Type Culture Collection (Manassas, VA) and maintained in maintained in DMEM-F12 (1∶1) supplemented with 10% FBS. Estradiol (E2) and 4-hydroxytamoxifen (4OHT) were purchased from Sigma-Aldrich (St. Louis, MO). The ERα-selective agonist propyl-pyrazole-triol (PPT), ERβ-selective agonist diarylpropionitrile (DPN) and the ER antagonist ICI 182780 (ICI) were purchased from TOCRIS (Ellisville, MO). The GPR30-specific agonist G1 was purchased from Merck KGaA (Darmstadt, Germany). MEK inhibitor U0126 was obtained from Promega (Madison, WI). The following antibodies were used: ERα (Bethyl Laboratories, Montgomery, TX); ERβ (Oncogene Research Products, San Diego, CA); phospho-p42/p44 ERK/MAPK (Cell Signaling, Beverly, MA); ERK1, ERK2, (Santa Cruz Biotechnology, Santa Cruz, CA); GPR30 (MBL International,Woburn,MA); hERα36 antibody was kind gift from Dr. ZhanoYi Wang (Creighton University Medical School, Omaha, NE).

### Cell extracts and immunoblotting

To prepare cell extracts, the cells were grown in 5% DCC medium for the period of 48 hrs and then treated with either E2 (10 nM), DPN (10 nM), PPT (10 nM) or G1 (10 nM). When indicated, ICI (1.0 µM) or 4OHT (1.0 µM) was added 1 hr before and MEK inhibitor U0126 (20 µM) was added 15 min before ligand treatment. The cells were washed once with PBS and then lysed in radioimmunoprecipitation assay buffer [50 mmol/l Tris-HCl (pH 7.5), 150 mmol/l NaCl, and 0.5% 1× protease inhibitor cocktail (Roche Applied Science, Indianapolis, IN)] for 10 minutes on ice. The cell lysates were centrifuged at 13,000 rpm for 10 min at 4°C. The cell lysates containing an equal amount of protein were then resolved on a sodium dodecyl sulfate-polyacrylamide gel (8% acrylamide), transferred to a nitrocellulose membrane, probed with the appropriate antibodies, and developed using the ECL detection reagent (Amersham Pharmacia Biosciences, Piscataway, NJ).

### Immunoflourescent labeling and confocal microscopy

The cellular localization of proteins was determined by indirect immunofluorescence labeling. SUM149 and SUM190 cells were grown on sterile glass coverslips in 5% DCC media for 48 hrs, fixed in 4% paraformaldehyde, permeabilized in 0.1% Triton X-100, and blocked in 5% normal goat serum-PBS. The cells were incubated with the primary antibodies for 1 hr, washed thrice in PBS, and then incubated with goat anti-mouse or goat anti-rabbit secondary antibodies conjugated with Alexa 488 from Molecular Probes (Eugene, OR). For actin cytoskeletal staining, phalloidin conjugated with Alexa 546 was used (Molecular Probes). The DNA dye DAPI was used as nuclear stain (blue).

### Matrigel invasion and migration assays

To quantify the cell migration potential of IBC cells, the cells were grown in 5% DCC media for 48 hrs. The cells were then trypsinised for collection, washed in PBS, resuspended in 0% DCC medium containing 0.1% BSA, and loaded into the upper well of an uncoated Boyden chamber (BD Biosciences) at a concentration of approximately 3.3×10^4^ cells/well. The lower side of the separating filter was filled 0.2% DCC medium containing 0.1% BSA. The cells were fixed after about 16 hrs and the cells which had successfully migrated were stained and counted manually. The same protocol was followed for measuring the invasion potential with principal differences being the use of Matrigel-coated Boyden chamber (BD Biosciences) and loading of 1×10^5^ cells/well.

### Immunohistochemistry

Deparaffinized sections were subjected to antigen retrieval by boiling the sections in 10 mM citric acid buffer (pH 6.0) for 10 min. Sections were then incubated with rabbit p-ERK1/2 antibody (Cell Signaling) at 4°C overnight, followed by incubation with EnVision (Dako, Carpinteria, CA) for 1 hour at room temperature. Immunostained sections were lightly counterstained with hematoxylin, dehydrated in graded ethanol, cleared in xylene, and mounted with the use of the peramount mounting medium.

## Supporting Information

Figure S1 **Phosphorylation of Akt, JNK and p38 upon E2 treatment in SUM149 cells.** The cells were maintained in 5% DCC for 48 hours and then treated with E2 (10 nM) and then Akt, JNK and p38 phosphorylation were analyzed by western blotting.
(TIF)

Figure S2 **Cell proliferation of SUM149 with E2 treatment.** The cells were plated in 6-well plates and treated with E2 the next day. The cell growth was determined by counting cell number using a Coulter Counter.
(TIF)
