# Supplementary figures and images for: Alternate Estrogen Receptors Promote Invasion of Inflammatory Breast Cancer Cells via Non-Genomic Signaling
Source: PLoS One. 2012 Jan 25;7(1):e30725. doi: 10.1371/journal.pone.0030725 (PMC3266301; doi:10.1371/journal.pone.0030725)

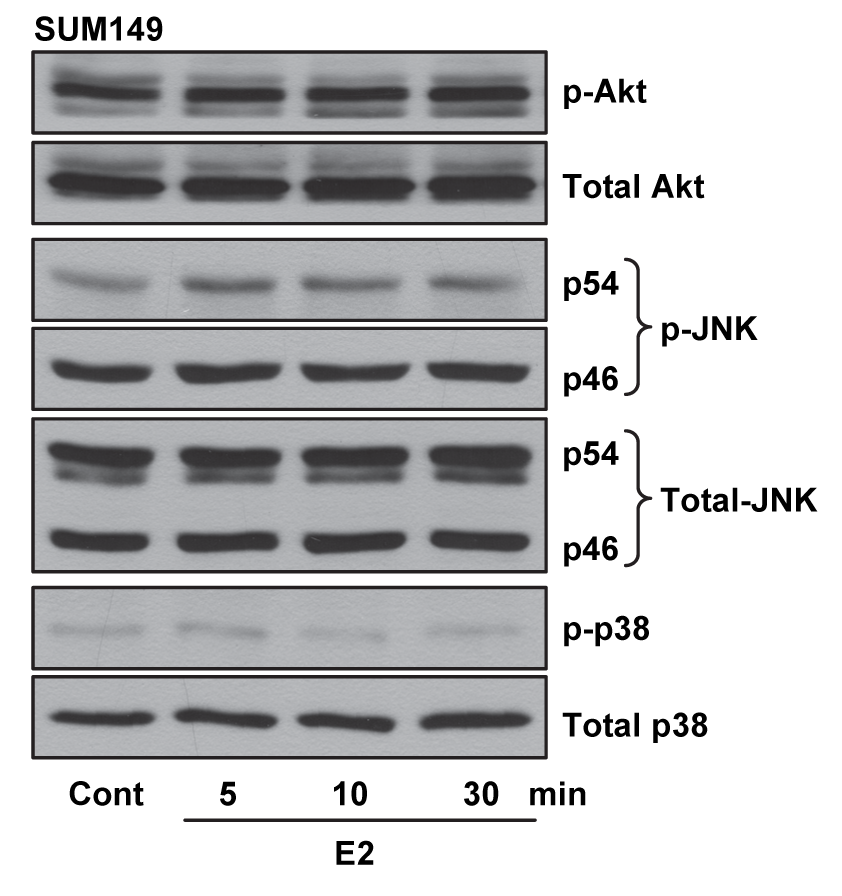

Supplement: Figure S1 — Phosphorylation of Akt, JNK and p38 upon E2 treatment in SUM149 cells. The cells were maintained in 5% DCC for 48 hours and then treated with E2 (10 nM) and then Akt, JNK and p38 phosphorylation were analyzed by western blotting. (TIF) [file pone.0030725.s001.tif]

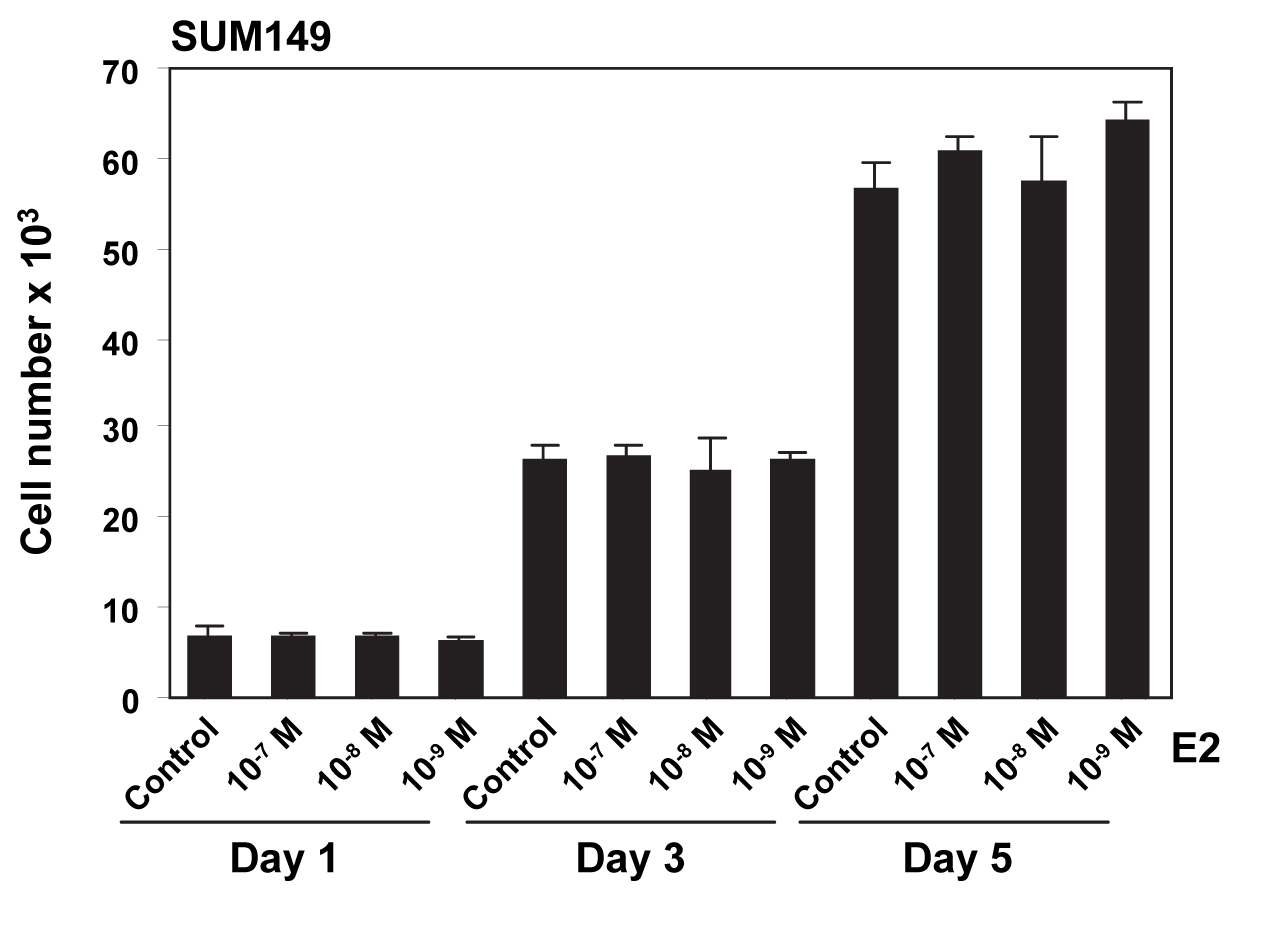

Supplement: Figure S2 — Cell proliferation of SUM149 with E2 treatment. The cells were plated in 6-well plates and treated with E2 the next day. The cell growth was determined by counting cell number using a Coulter Counter. (TIF) [file pone.0030725.s002.tif]
